# Molecular diversity of bacteria in commercially available “Spirulina” food supplements

**DOI:** 10.7717/peerj.1610

**Published:** 2016-01-21

**Authors:** Elisabeth Vardaka, Konstantinos A. Kormas, Matina Katsiapi, Savvas Genitsaris, Maria Moustaka-Gouni

**Affiliations:** 1 Department of Nutrition and Dietetics, Alexander Technological Educational Institute of Thessaloniki, Thessaloniki, Greece; 2 Department of Ichthyology and Aquatic Environment, University of Thessaly, Volos, Greece; 3 School of Biology, Aristotle University of Thessaloniki, Thessaloniki, Greece

**Keywords:** *Arthrospira*, *Spirulina*, Food supplements, Cyanobacteria, Bacteria, Pyrosequencing

## Abstract

The cyanobacterium *Arthrospira* is among the most well-known food supplements worldwide known as “Spirulina.” While it is a widely recognized health-promoting natural product, there are no reports on the molecular diversity of commercially available brands of “Spirulina” supplements and the occurrence of other cyanobacterial and heterotrophic bacterial microorganisms in these products. In this study, 454-pyrosequencing analysis of the total bacterial occurrence in 31 brands of “Spirulina” dietary supplements from the Greek market was applied for the first time. In all samples, operational taxonomic units (OTUs) of *Arthrospira platensis* were the predominant cyanobacteria. Some products contained additional cyanobacterial OTUs including a few known potentially toxic taxa. Moreover, 469 OTUs were detected in all 31 products collectively, with most of them being related to the Proteobacteria, Firmicutes, Bacteroidetes, Actinobacteria and Verrucomicrobia. All samples included heterotrophic bacterial OTUs, ranging from 9–157 per product. Among the most common OTUs were ones closely related to taxa known for causing health issues (i.e., *Pseudomonas*, *Flavobacterium*, *Vibrio*, *Aeromonas*, *Clostridium*, *Bacillus*, *Fusobacterium*, *Enterococcus*). The observed high cyanobacterial and heterotrophic bacterial OTUs richness in the final product is a point for further research on the growth and processing of *Arthrospira* biomass for commercial purposes.

## Introduction

In the present health food market, the filamentous cyanobacterium *Arthrospira*, has been widely used as a dietary supplement under the usual commercial designation “Spirulina,” due to its high nutritional value (e.g., high quantities of proteins, large amounts of essential fatty acids, polysaccharide, vitamins, minerals, and pigments) and its putative beneficial health effect (e.g., antioxidant, antiviral, anticancer activity), (Becker, 1994; Gantar & Svirčev, 2008; Gershwin & Belay, 2008; Hu, 2004).

*Arthrospira* is usually presented under the taxonomically incorrect name *Spirulina* (Vonshak & Tomaselli, 2000). Since 1852, the classification of the genus *Spirulina,* based mainly on morphological features, has been a subject of long debate between taxonomists. In the last decades of the 20th century, new information have drastically changed the criteria that are used for the taxonomic classification of cyanobacteria (Castenholz, 1989; Komárek & Anagnostidis, 2005). According to the current polyphasic approach, which combines morphological, cytological, ecological, biochemical and molecular criteria, the genus *Spirulina* has been re-evaluated, leading to the recognition of two separated genera, *Arthrospira* and *Spirulina* (Komárek & Anagnostidis, 2005). Although, the name *Arthrospira* has become universally accepted for the cultivated species, further clarification of their systematic position in the genus *Arthrospira* is still ongoing. Komárek (2016), in his revision on the taxonomy and nomenclature of *Arthrospira* species, has recommended the replacement of the commercial species name *Arthrospira platensis*, with the taxonomically-correct name of *Arthrospira fusiformis*. The complicated and still debated taxonomy of *Arthrospira* and its relationship with *Spirulina* raises serious concerns regarding the “identity” of traditionally edible cyanobacteria and as a consequence, regarding their nutritional quality (e.g., unlike *Spirulina*, *Arthrospira* contains the unsaturated fatty acid *γ*-linolenic acid) and the safety (e.g., unlike *Spirulina*, *Arthrospira* is known to be potential toxin producer) of their products (Ballot et al., 2004; Gantar & Svirčev, 2008; Lugomela, Pratap & Mgay, 2006; Vonshak & Tomaselli, 2000).

The growing consumers’ demand for cyanobacterial products as dietary supplements has offered the opportunity for rapidly growing commercial cultivation of *Arthrospira* all over the world. Since *Arthrospira* has an optimum growth temperature in the range of 35–38°C, large-scale cultivation is mainly located in tropical, sub-tropical and warm temperate climate zones. Principal producers such as Earthrise Nutritionals in California and Cyanotech Corporation of Hawaii, USA; Hainan DIC Marketing in Hainan Island, China and Siam Algae Company in Bangkok, Thailand, produce together about 1,300 t (dw) of *Arthrospira* annually (Gershwin & Belay, 2008). Today, China seems to be the most actively engaged country in cultivating *Arthrospira*, aiming to reach annual production to 10^6^ t (dw) (Lu, Xiang & Wen, 2011).

The biomass of *Arthrospira* used for commercial exploitation as part of the human diet, is produced nearly exclusively in outdoor open systems, either obtained through a controlled cultivation process in open raceway ponds or harvested from natural environments (Ballot et al., 2004; Becker, 1994; Grewe & Pulz, 2011; Hu, 2004; Li & Qi, 1997). Outdoor closed systems using greenhouses ponds have been introduced in mid latitude areas (e.g., China and Europe) in order to rise the production period per year (Lu, Xiang & Wen, 2011; E Vardaka & KA Kormas, pers. obs., 2015). Advanced technology of closed photobioreactors is being successfully implemented to commercially produce *Arthrospira* (e.g., in Ritschenhausen, Thuringia, Germany); however, their use is very limited probably due to the high construction and operation cost (Grewe & Pulz, 2011).The main limitation of outdoor open/closed systems seems to be the risk of contamination by fungi, bacteria and protozoa, and competition by other cyanobacteria and microalgae that tend to dominate, regardless the original species used as inoculum (Tredici, 2004). This risk is much higher in natural environments, where the biomass harvested is essentially a mixture of multiple species of cyanobacteria and other microorganisms (Carmichael, Drapeau & Anderson, 2000). These challenges can be magnified as processes are scaled up (harvesting, drying and packaging) affecting the final quality of the product.

Although there is an existing threat concerning the involuntary inclusion of microbial contaminants in the dietary supplements, which may include potentially toxin-producing cyanobacteria or unwanted pathogens, to the best of our knowledge, no reports on the microbial content of such supplements have been published, based on sequencing methodologies. The present study serves as a “first step” to assess possible bacterial contamination of “Spirulina” supplements and consequently to allow for further studies and a stricter monitoring of these products. Thus, we applied 454-pyrosequencing analysis of the 16S rRNA gene in order to investigate whether commercially available brands of “Spirulina” supplements in the Greek market–with most of them having international commercial circulation- contain other prokaryotes in addition to *Spirulina*/*Arthrospira*, and to characterize prokaryotic phylotype richness of these products for the first time. Since these supplements are directly and largely consumed by the public, the presence of any non-target microorganism is of importance as a possible source of microbial contamination.

## Materials and Methods

### Sample collection and handling

A total of 31 “Spirulina” dietary supplements of different brands were obtained from internet distributors, pharmacies and health food retail in Greece in 2013. Based upon the product labels and/or website information, most of the “Spirulina” products, originate from controlled cultures (open ponds) of different geographical origin (Europe, Asia, USA and Australia) and have international selling distribution network (Table 1). Four of the products come from greenhouse cultured ponds that operate in Greece. The most common species name referred in the labels of “Spirulina” dietary supplements is *Arthrospira* (*Spirulina*) *platensis* (16/31), while 15/31 products were labeled as containing “Spirulina.”

**Table 1:** Commercially available “Spirulina” food supplements from the Greek market. OTUs: bacterial operational taxonomic units.

Product code	Product type	Origin of manufacturing company^*^	Cultivation system^*^	Cyanobacteria listed on the product label	Cyanobacterial OTUs richness	Heterotrophic bacterial OTUs richness
SP1	Capsule	Greece	Greenhouse pond	“Spirulina”	3	96
SP2	Tablet	Germany	–	“Spirulina”	3	91
SP3	Tablet	Greece	Greenhouse pond	“Spirulina”	4	119
SP4	Powder (raw)	–	–	“Spirulina”	1	15
SP5	Capsule	India	–	“Spirulina”	3	73
SP6	Powder	Greece	Greenhouse pond	*Spirulina platensi*s	1	78
SP7	Tablet	–	–	“Spirulina”	1	29
SP8	Tablet	Taiwan	Open pond	*Spirulina platensis*	1	52
SP9	Tablet	–	–	*Arthrospira platensis*	1	84
SP10	Tablet	Hawaii	Open pond	*Arthrospira platensis*	3	156
SP11	Tablet	–	–	*Arthrospira platensis*	3	109
SP12	Tablet	Germany	–	*Spirulina platensis*	1	10
SP13	Tablet	Hawaii	Open pond	*Arthrospira platensis*	6	95
SP14	Tablet	USA	–	“Spirulina”	1	39
SP15	Capsule	India	Open pond	*Arthrospira platensis*	1	101
SP16	Tablet	–	–	*Arthrospira platensis*	2	84
SP17	Tablet	Australia	–	*Arthrospira platensis*	2	16
SP18	Tablet	Hawaii	–	*Spirulina platensis*	1	48
SP19	Tablet	Italy	–	*Arthrospira platensis*	4	133
SP20	Capsule	France	–	*Spirulina platensis*	1	36
SP21	Tablet	Germany	Open pond	“Spirulina”	2	88
SP22	Capsule	Greece	–	Spirulina	2	102
SP23	Powder (raw)	–	–	“Spirulina”	1	118
SP24	Tablet	UK	–	*Arthrospira platensis*	2	104
SP25	Tablet	Greece	Greenhouse pond	“Spirulina”	3	157
SP26	Tablet	–	–	Spirulina	1	76
SP27	Tablet	Cuba	Open pond	*Spirulina platensis*	1	114
SP28	Capsule	Australia	–	*Spirulina platensis*	3	75
SP29	Drops	Europe	–	“Spirulina”	3	37
SP30	Candies	Europe	–	“Spirulina”	4	68
SP31	Bar	Germany	–	“Spirulina”	1	9

**Notes.**

* Where (–), no information available.

“Spirulina” samples were in the form of tablets (19/31), capsules (6/31), powders (3/31), candies (1/31), drops (1/31) and bar (1/31) (Table 1). Capsules were aseptically removed from the capsular form of samples before further analysis. Tablets, powders, candies and bar forms were homogenized aseptically using a mortar and pestle. Approximately, 100 mg of each sample or a maximum of 10 drops was used for DNA extraction. Each sample for the DNA extraction consisted of a pooled triplicate sample. Three tablets/capsules/powder/candies from the same product batch were pooled together while for the liquid sample three bottles were mixed together before sampling the 10 drops.

### Molecular analysis

DNA was extracted using the PowerMax Soil DNA Isolation kit (MoBio, Carlsbad, CA, USA) according to manufacturer’s protocol. Three blank DNA extractions were included in order to test microbial contamination of the kit; no amplifiable DNA was detected in these analysis. Tag-pyrosequencing of the 16S rRNA gene was performed using PCR amplification of the V4-V6 region of the 16S rRNA gene and the primer pair S-DBact-0341-b-S-17 (5′-CCTACGGGNGGCWGCAG-3′) and S-D-Bact-0785-a-A-21 (5′-GACTACHVGGGTATCTAATCC-3′) for bacteria (Klindworth et al., 2013). Sequencing was performed as described in Dowd et al. (2008) in Roche 454 FLX titanium instruments and reagents after following manufacturer’s guidelines at the MRDNA Ltd. (Shallowater, TX, USA) sequencing facilities. Data processing and quality control were performed with the MOTHUR software (v 1.30) (Schloss et al., 2009) including denoising of the flowgrams using PyroNoise (Quince et al., 2009). Sequences with ≥250 bp and no ambiguous or no homopolymers ≥8 bp were included for further analysis. These sequences were aligned using the SILVA SSU database (release 108, Pruesse et al., 2007). All sequences were binned into Operational Taxonomic Units (OTUs) and were clustered (average neighbor algorithm) at 97% sequence similarity (Kunin et al., 2010; Stackebrandt & Goebel, 1994). Taxonomic classification was based on the SILVA 108 database. The batch of sequences from this study has been submitted to the Short Reads Archive (http://www.ncbi.nlm.nih.gov/sra) accession number SRR2057094.

### Statistical analysis

Since the heterotrophic bacterial OTUs richness was high in the 31 “Spirulina” dietary supplements samples, and the origin and handling process of each product were not obvious, the samples were grouped with cluster analysis (Sokal & Rohlf, 1981) using the Bray-Curtis similarity index in log transformed relative abundance values of the heterotrophic bacterial OTUs in order to investigate the differences between the samples attributed to these OTUs. The analysis was performed using the PAST software (Hammer, Harper & Ryan, 2001).

## Results and Discussion

In this study, we aimed at depicting whether the cyanobacterial biomass of 31 commercial supplements available in the Greek market (a) are dominated by *Spirulina*/*Arthrospira* spp. and (b) they include other heterotrophic Bacteria, possible originating from the production line of the supplements. For this purpose, we applied 454-pyrosequencing analysis of cyanobacterial and total bacterial occurrence in these 31 products as one of the most inclusive tools in revealing the presence of known and yet-uncultivated bacteria. Although the limitations of the 454-pyrosequencing approach are well-known (e.g., limited primer universality (Hadziavdic et al., 2014), PCR amplification errors, nucleotide misincorporation, PCR chimera formation (Stoeck et al., 2010; Pawlowski et al., 2011), pyrosequencing errors (Kunin et al., 2010)), it can provide a complete picture of the diversity in such samples on the OTU level.

**Figure 1:**
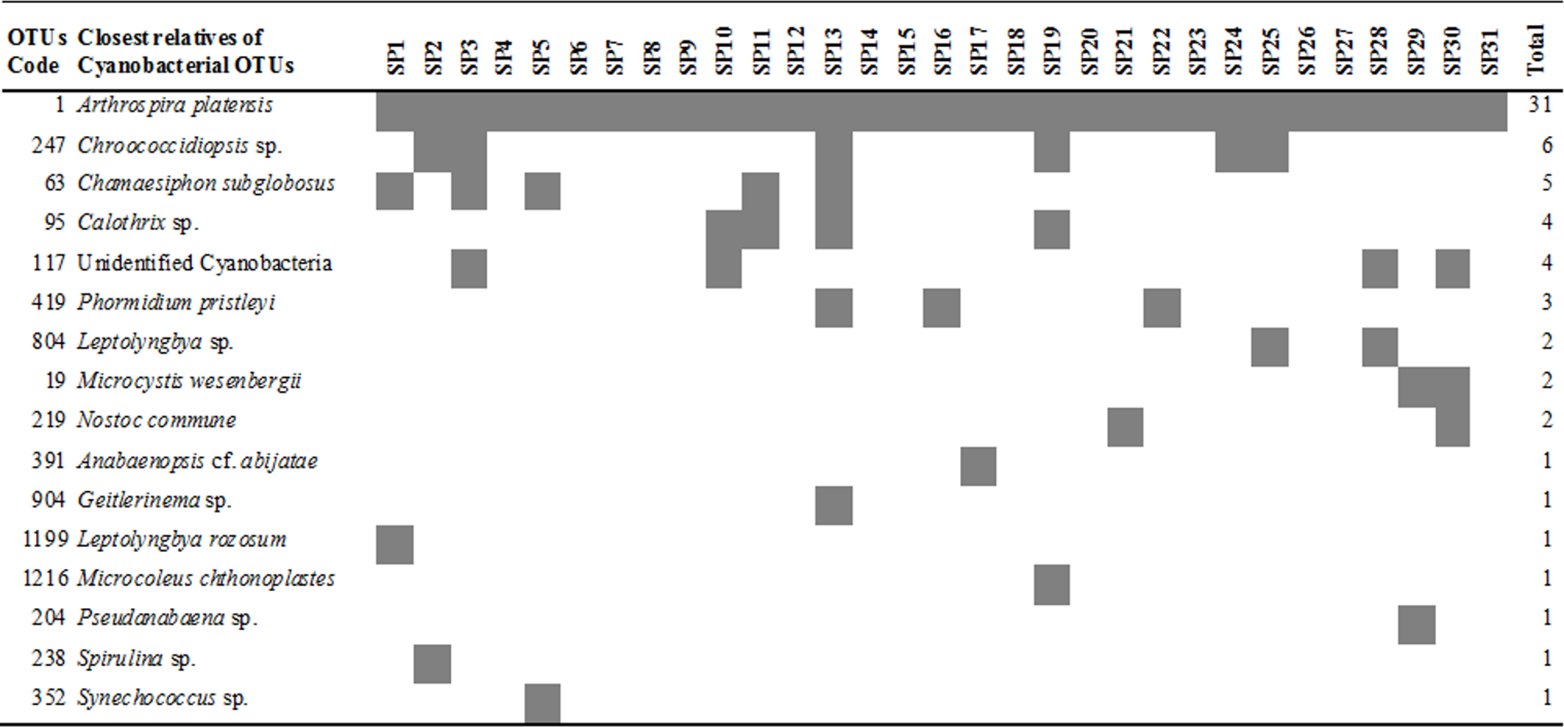
Heatmap of occurrence of the operational taxonomic units (OTUs) that were closely related to cyanobacteria in commercially available “Spirulina” food supplements in the Greek market.

### Cyanobacteria in “Spirulina” products

All of the *Arthrospira*-related OTUs which were found in the commercially available “Spirulina” food supplements in the Greek market, were related only to *Arthrospira platensis* (Fig. 1), which was recently revised by Komárek (2016) as *Arthrospira fusiformis*. In each sample, *A. platensis* (*A. fusiformis*) was clearly the predominant taxon (81.2–100.0%) among the Cyanobacteria in all but the product SP29, in liquid form (Table 1), in which *Arthrospira* spp. comprised only 48.6% and co-dominated with *Microcystis wesenbergii*-related OTUs (48.3%).

Although *Arthrospira* is growing at selective culture growth media (e.g., alkaline conditions, high salt concentrations) that do not allow the growth of most microorganisms, it can be subject to competition by cyanobacteria belonging to different genera (Becker, 1994). In our study, 13/31 products contained only *A. platensis* while in the rest 18 products an additional one to five cyanobacteria were found (Fig. 1). The most commonly found non-*Arthrospira* OTUs were related to *Chroococcidiopsis* sp. (6/31) followed by *Chamaesiphon subglobosus* (5/31), *Calothrix* sp. (4/31), *Phormidium pristleyi* (4/31), *Microcystis wesenbergii* (2/31), *Nostoc commune* (2/31), *Leptolyngbya* sp. (2/31), *Anabaenopsis* cf. *abijatae* (1/31), *Pseudanabaena* sp. (1/31), *Geitlerinema* sp. (1/31), *Leptolyngbya rozosum* (1/31), *Microcoleus chthonoplastes* (1/31) and *Spirulina* sp. (1/31). *Microcystis, Nostoc* and *Anabaenopsis* species are known as potentially toxic producing cyanobacteria. Toxin production by cyanobacteria is species- and strain-specific and depends on environmental conditions (Sivonen & Jones, 1999), thus the presence of cyanotoxins cannot be predicted in this study.

**Figure 2:**
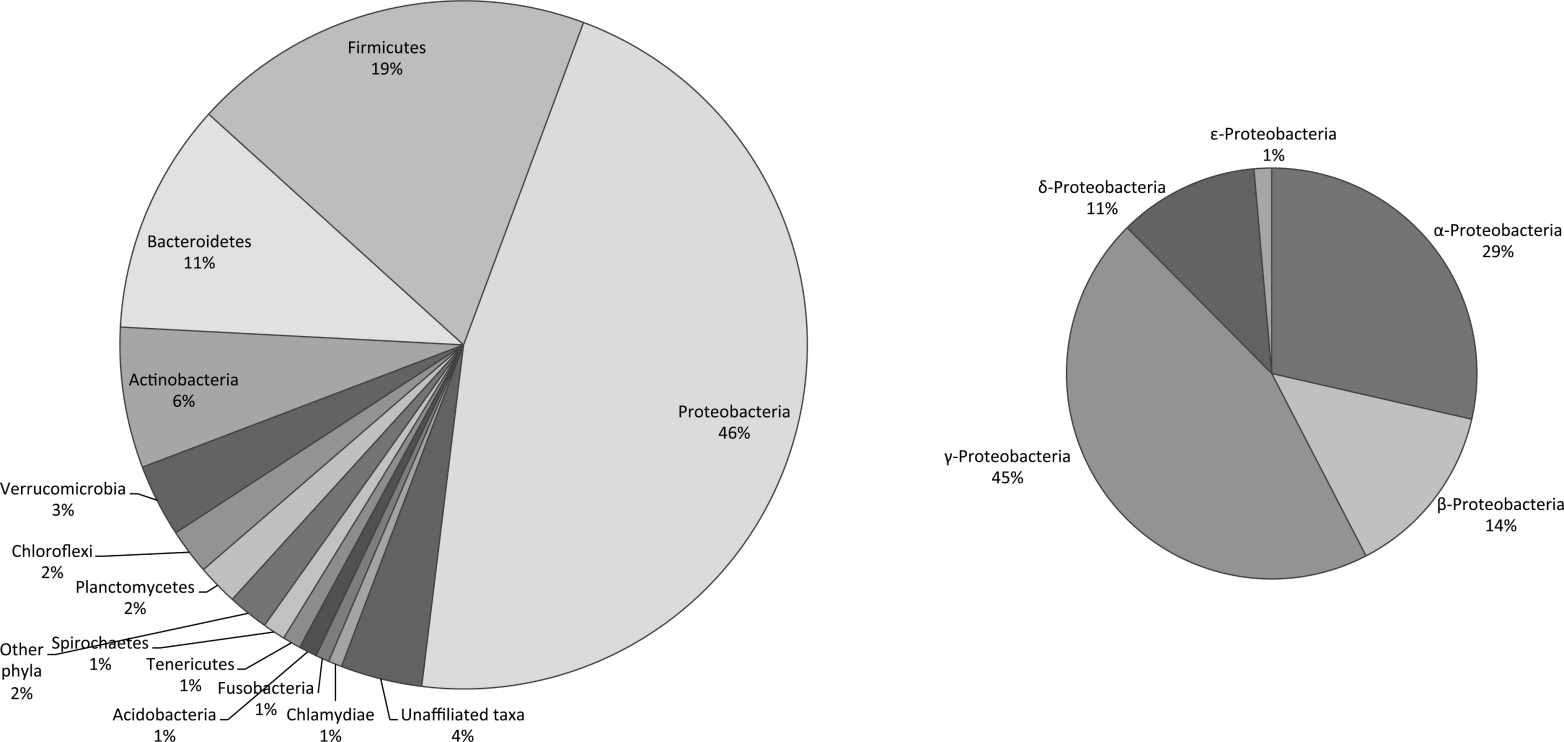
Relative richness of the heterotrophic bacterial operational taxonomic units (OTUs) at the phylum level, found in commercially available “Spirulina” food supplements in the Greek market.

**Figure 3:**
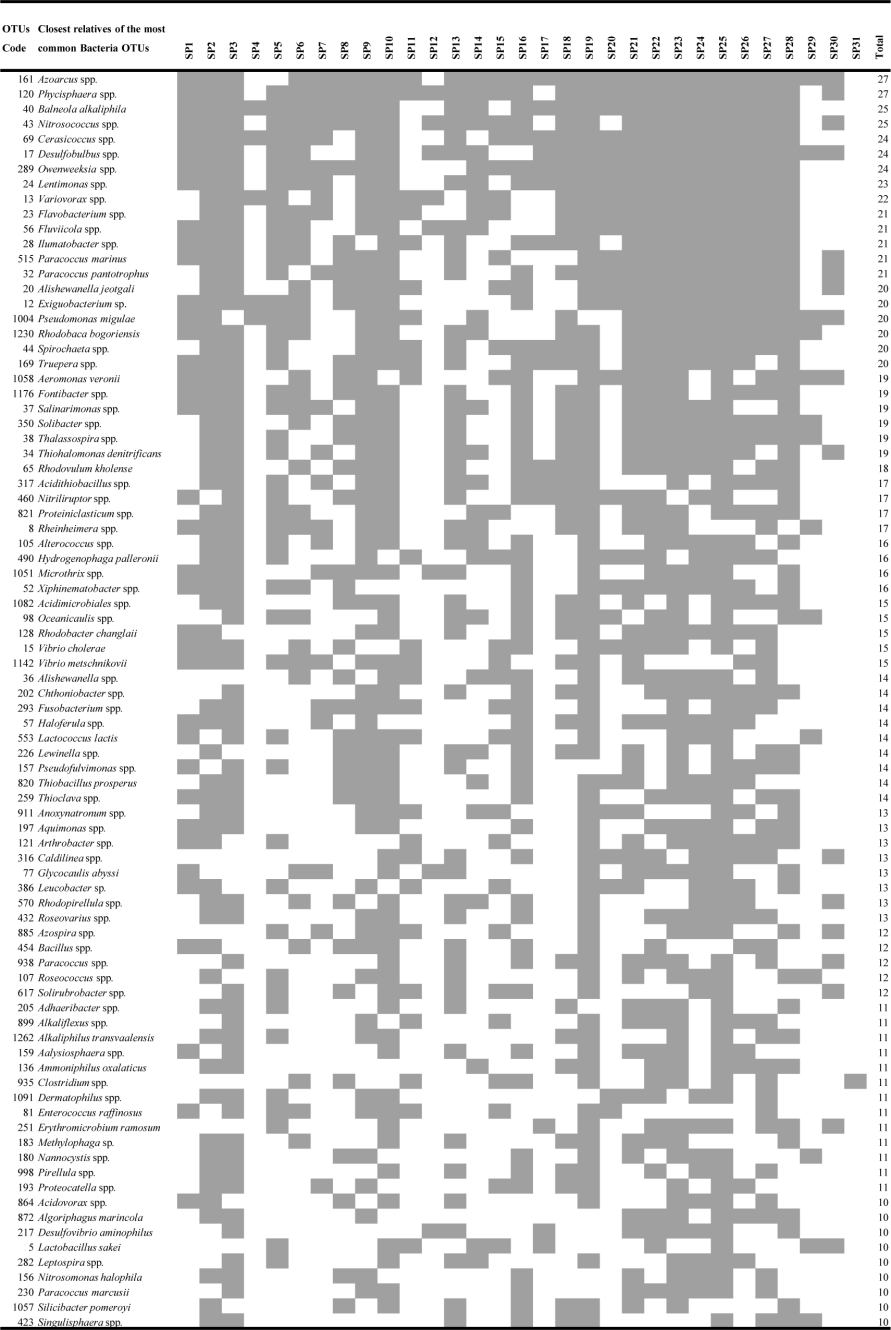
Heatmap of occurrence of the most common (found in ≥10 products) operational taxonomic units (OTUs) that were closely related to heterotrophic bacteria in commercially available “Spirulina” food supplements in the Greek market.

### Heterotrophic bacterial DNA in “Spirulina” products

In non-sterile large-scale cultivation systems of cyanobacteria, substances released from cells or decomposition of dead cells, provide a source of organic compounds for attracting heterotrophic bacteria (Becker, 1994). The genetic imprint of microbial DNA in the final “Spirulina” product may reflect the occurrence of bacteria in the different processing steps, i.e., culturing, harvesting, drying and packaging, of *Arthrospira* commercial production facilities. In our study, the bacterial OTU richness associated with “Spirulina” food supplements was high. A total of 469 unique heterotrophic bacterial OTUs were found in the 31 products. The unique heterotrophic bacterial OTUs belonged to 20 different phyla, while 18 OTUs could not be affiliated to any of the known taxa. The majority of the OTUs were members of five phyla: Proteobacteria (46% of the total number of OTUs), Firmicutes (19%), Bacteroidetes (11%), Actinobacteria (6%) and Verrucomicrobia (3%). Within the most diverse phylum (Proteobacteria), the classes of *γ*- and *α*-Proteobacteria dominated in terms of OTUs richness (45 and 29% of the Proteobacteria-related OTUs, respectively) (Fig. 2).

Heterotrophic bacterial OTUs richness, ranged from 9–157 per product. Ten products had >100 heterotrophic bacterial OTUs (Table 1). The 50% of OTUs were found to occur only in one or two “Spirulina” products, while the other 50% were found to be present at ≥3–27 of the products. Among the most common OTUs, present in ≥10 products, OTUs closely related to *Azoarcus* (27/31), *Phycisphaera* (27/31), *Balneola* (25/31), *Nitrosococcus* (25/31) were detected (Fig. 3). Although potential pathogenicity of the present heterotrophic bacterial OTUs cannot be proved with the length of the produced reads, the fact that among the most common OTUs were ones closely related to taxa known for causing health issues (i.e., *Pseudomonas*, *Flavobacterium, Vibrio, Aeromonas, Clostridium, Bacillus, Fusobacterium, Enterococcus*; Fig. 3) shows the need for stricter monitoring of these supplements. For example, some *Bacillus* spp. seem to cause health problems when found in nutritional supplements (Stickel et al., 2009). In a recent study, potentially pathogenic *Clostridium* spp. from commercial *Arthrospira* products that were negative for faecal coliform tests were isolated (Hoekstra et al., 2011). Moreover, the presence of pathogens in *Arthospira* products raises the questions whether some of the sporadic cases of health effects in humans which have been reported after the consumption of food supplements (e.g., Iwasa et al., 2002; Mazokopakis et al., 2008) are due to the *Arthropsira* itself or its contained bacteria (Hoekstra et al., 2011; Warburton et al., 1998). However, whether these microorganisms are viable, and thus potentially pathogenic, requires further investigation. Further testing is also required to evaluate any idea of either nutritional value or public health risk or both; among them, the measurement of vitamins, trace element levels, and toxins. Toxin occurrence screening and cytotoxicity assays of the samples of this study are underway and will be reported elsewhere.

The majority of the heterotrophic bacterial OTUs in our samples were closely related to microorganisms usually found in aquatic and terrestrial habitats, and waste and wastewater treatment systems. Moreover, a part of the detected OTUs were closely related to animal and human microbiota (e.g., skin, gut). This may indicate that water-attracted animals (e.g., birds, rodents) and humans when handling the product during the different processing steps (e.g., harvesting, drying) may be a point of microbial contamination.

**Figure 4:**
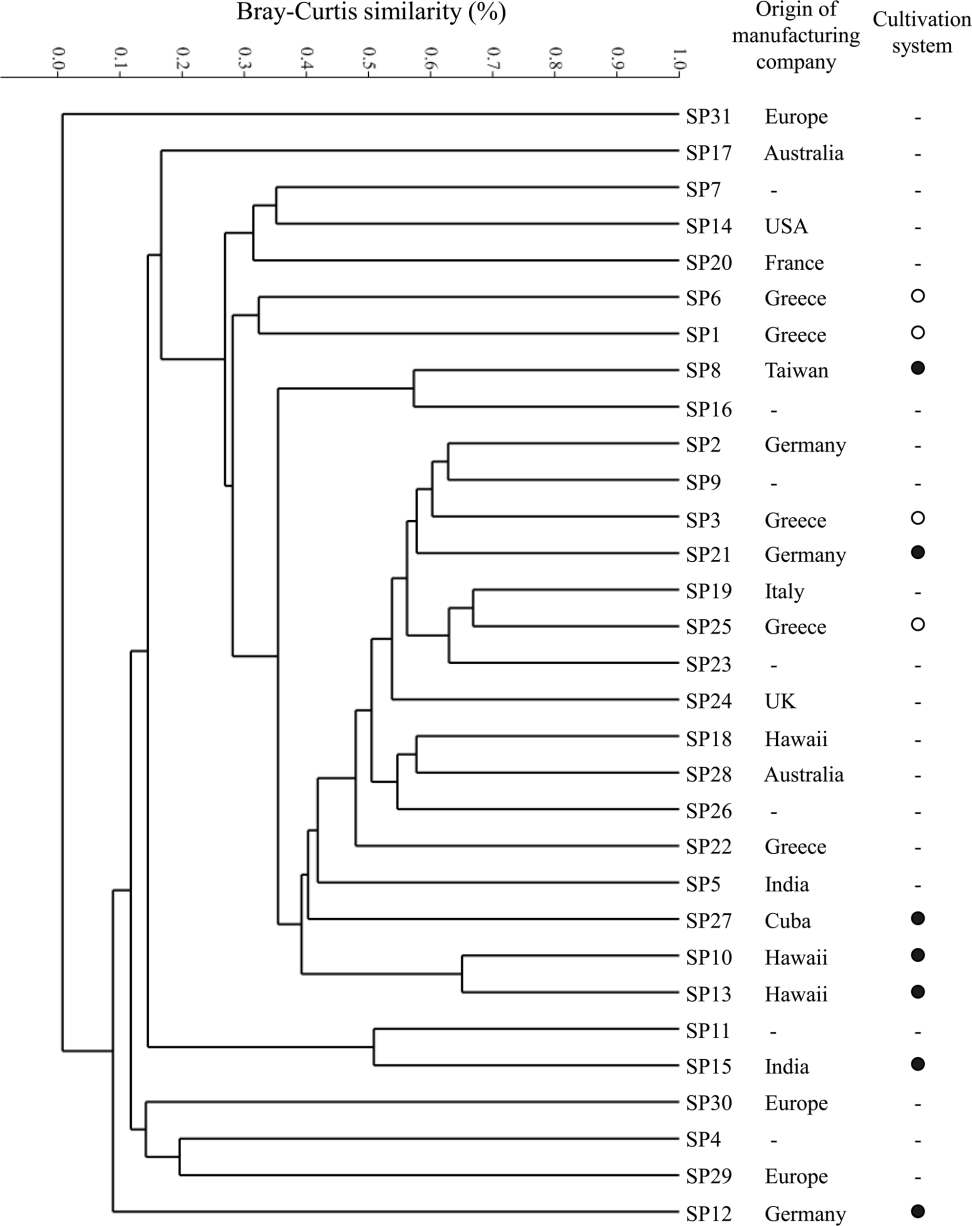
Cluster analysis based on the Bray-Curtis similarity index in log transformed relative abundance values of the heterotrophic bacterial OTUs found in the 31 “Spirulina” dietary supplements (SP1-SP31) from the Greek market. For each sample, the geographical origin of the manufacturing company and the type of cultivation system used (open: ●, greenhouse: ○) are also given based on the available information from the product labels and/or their websites. Where (-): no information is available.

According to cluster analysis and based on the limited available information from the product labels and/or their websites, it seems that the occurrence of heterotrophic bacterial OTUs in our samples is not associated with the geographical origin of the manufacturing company or the type of cultivation system used (Fig. 4). Cluster analysis revealed that samples SP31, SP12, SP29, SP4 and SP30 were the most different ones. All these samples were among the ones with the lowest OTU richness (Table 1). Moreover, samples SP31, SP30 and SP29 were the only ones which were not in the form of pills or powder (Table 1), and for this they are expected to include several other ingredients but also different and more complex preparation process which might increase the microbial burden of the product.

## Conclusions

In conclusion, this study showed that although the dominant cyanobacterium in the 31 commercially available “Spirulina” products of the Greek market, is *Arthropsira* spp., several of these products contain other cyanobacteria as well. Moreover, more than 450 heterotrophic bacterial OTUs were found across the 31 products, with varying OTUs richness and abundance. Although this study did not aim at the investigation of the bacterial cells viability, the unexpected high cyanobacterial and heterotrophic bacterial OTUs richness detected in some of the products raises the demand for stricter monitoring and improvements in the commercial growth conditions of *Arthrospira* biomass and its production processes as a food supplement.

## Additional Information and Declarations

### Data Availability

The following information was supplied regarding data availability: The only raw data produced by this study have been deposited to the Short Reads Archive (http://www.ncbi.nlm.nih.gov/sra) accession number SRR2057094.

## References

[ref-1] Ballot A, Krienitz L, Kotut K, Wiegand C, Metcalf JS (2004). Cyanobacteria and cyanobacterial toxins in three alkaline Rift Valley lakes of Kenya—Lakes Bogoria, Nakuru and Elmenteita. Journal of Plankton Research.

[ref-2] Becker EW (1994). Microalgae: biotechnology and microbiology.

[ref-3] Carmichael WW, Drapeau C, Anderson DM (2000). Harvesting of *Aphanizomenon flos*-*aquae* Ralfs ex Born and Flash. var. *flos-aquae* (cyanobacteria) from Klamath Lake for human dietary use. Journal of Applied Phycology.

[ref-4] Castenholz RW, Staley JT, Bryant MP, Pfenning N, Holt JG (1989). Subsection III, Order Oscillatoriales. Bergey’s manual of systematic bacteriology.

[ref-5] Dowd S, Callaway T, Wolcott R, Sun Y, McKeehan T, Hagevoort R, Edrington TS (2008). Evaluation of the bacterial diversity in the feces of cattle using 16S rDNA bacterial tag-encoded FLX amplicon pyrosequencing (bTEFAP). BMC Microbiology.

[ref-6] Gantar M, Svirčev Z (2008). Microalgae and cyanobacteria: food for thought. Journal of Phycology.

[ref-7] Gershwin ME, Belay A (2008). Spirulina in human nutrition and health.

[ref-8] Grewe CB, Pulz O, Whitton BA (2011). The biotechnology of cyanobacteria. Ecology of cyanobacteria II: their diversity in space and time.

[ref-9] Hadziavdic K, Lekang K, Lanzen A, Jonassen I, Thompson EM, Troedsson C (2014). Characterization of the 18S rRNA gene for designing universal eukaryote specific primers. PLoS ONE.

[ref-10] Hammer Ø, Harper D, Ryan P (2001). PAST: paleontological statistics software package for education and data analysis. Palaeontologia Electronica.

[ref-11] Hoekstra DT, Volschenk H, Collins M, McMaster LD (2011). An investigation of *Clostridium* species present in nutraceutical preparations of *Arthrospira platensis* (*Spirulina*) for human consumption. Journal of Applied Phycology.

[ref-12] Hu Q, Richmond A (2004). Industrial production of microalgal cell-mass and secondary products-Major industrial species: *Arthrospira* (*Spirulina*) *platensis*. Handbook of microalgal culture. Biotechnology and applied phycology.

[ref-13] Iwasa M, Yamamoto M, Tanaka Y, Kaito M, Adachi Y (2002). Spirulina-associated hepatotoxicity. American Journal of Gastroenterology.

[ref-14] Klindworth A, Pruesse E, Schweer T, Peplies J, Quast C, Horn M, Glöckner FO (2013). Evaluation of general 16S ribosomal RNA gene PCR primers for classical and next-generation sequencing-based diversity studies. Nucleic Acids Research.

[ref-15] Komárek J (2016). Review of the cyanobacterial genera implying planktic species after recent taxonomic revisions according to polyphasic methods: state as of 2014. Hydrobiologia.

[ref-16] Komárek J, Anagnostidis K (2005). Cyanoprocaryota. 2. Teil: Oscillatoriales.

[ref-17] Kunin V, Engelbrektson A, Ochman H, Hugenholtz P (2010). Wrinkles in the rare biosphere: pyrosequencing errors can lead to artificial inflation of diversity estimates. Environmental Microbiology.

[ref-18] Li DM, Qi YZ (1997). *Spirulina* industry in China: present status and future prospects. Journal of Applied Phycology.

[ref-19] Lugomela C, Pratap HB, Mgay YD (2006). Cyanobacteria blooms-a possible cause of mass mortality of Lesser Flamingos in Lake Manyara and Lake Big Momela, Tanzania. Harmful Algae.

[ref-20] Lu Y-M, Xiang W-Z, Wen Y-H (2011). *Spirulina* (*Arthrospira*) industry in Inner Mongolia of China: current status and prospects. Journal of Applied Phycology.

[ref-21] Mazokopakis EE, Karefilakis CM, Tsartsalis AN, Milkas AN, Ganotakis ES (2008). Acute rhabdomyolysis caused by Spirulina (*Arthrospira platensis*). Phytomedicine.

[ref-22] Pawlowski J, Christen R, Lecroq B, Bachar D, Shahbazkia HR, Amaral-Zettler L, Guillou L (2011). Eukaryotic richness in the abyss: insights from pyrotag sequencing. PLoS ONE.

[ref-23] Pruesse E, Quast C, Knittel K, Fuchs B, Ludwig W, Peplies J, Glöckner FO (2007). SILVA: a comprehensive online resource for quality checked and aligned ribosomal RNA sequence data compatible with ARB. Nucleic Acids Research.

[ref-24] Quince C, Lanzen A, Curtis TP, Davenport RJ, Hall N, Head IM, Read LF, Sloan WT (2009). Accurate determination of microbial diversity from 454 pyrosequencing data. Nature Methods.

[ref-25] Schloss PD, Westcott SL, Ryabin T, Hall JR, Hartmann M, Hollister EB, Lesniewski RA, Oakley BB, Parks DH, Robinson CJ, Sahl JW, Stres B, Thallinger GG, Van Horn DJ, Weber CF (2009). Introducing mothur: open-source, platform-independent, community-supported software for describing and comparing microbial communities. Applied and Environmental Microbiology.

[ref-26] Sivonen K, Jones G, Chorus I, Bartram J (1999). Cyanobacterial toxins. Toxic cyanobacteria in water.

[ref-27] Sokal RR, Rohlf FJ (1981). Biometry.

[ref-28] Stackebrandt E, Goebel BM (1994). Taxonomic note: a place for DNA:DNA reassociation and 16S rRNA sequence analysis in the present species definition in bacteria. International journal of systematic bacteriology.

[ref-29] Stickel F, Droz S, Patsenker E, Bögli-Stuber K, Aebi B, Leib SL (2009). Severe hepatotoxicity following ingestion of Herbalife^®^ nutritional supplements contaminated with *Bacillus subtilis*. Journal of Hepatology.

[ref-30] Stoeck T, Bass D, Nebel M, Christen R, Jones MD, Breiner HW, Richards TA (2010). Multiple marker parallel tag environmental DNA sequencing reveals a highly complex eukaryotic community in marine anoxic water. Molecular Ecology.

[ref-31] Tredici MR, Richmond A (2004). Mass production of microalgae: photobioreactors. Handbook of microalgal culture. Biotechnology and applied phycology.

[ref-32] Vonshak A, Tomaselli L, Whitton BA, Potts M (2000). *Arthrospira* (*Spirulina*): systematics and ecophysiology. The ecology of Cyanobacteria. Their diversity in time and Space.

[ref-33] Warburton DW, Harrison B, Crawford C, Foster R, Fox C, Gour L, Purvis U (1998). Current microbiological status of health foods sold in Canada. International Journal of Food Microbiology.

